# Relation of Chemical Composition and Colour of Spruce Wood

**DOI:** 10.3390/polym14235333

**Published:** 2022-12-06

**Authors:** Viera Kučerová, Richard Hrčka, Tatiana Hýrošová

**Affiliations:** 1 Department of Chemistry and Chemical Technologies, Faculty of Wood Sciences and Technology, Technical University in Zvolen, T.G. Masaryka 24, 96001 Zvolen, Slovakia; 2 Department of Wood Sciences, Faculty of Wood Sciences and Technology, Technical University in Zvolen, T.G. Masaryka 24, 96001 Zvolen, Slovakia; 3 Department of Mathematics and Descriptive Geometry, Faculty of Wood Sciences and Technology, Technical University in Zvolen, T.G. Masaryka 24, 96001 Zvolen, Slovakia

**Keywords:** spruce wood, chemical composition, anatomical section, colour

## Abstract

The visual inspection of fresh cut spruce wood (*Picea abies*, L. Karst.) showed the variability of its colour. Wood visual inspection is a part of wood quality assessment, for example, prior to or after its processing. The detail spruce wood colour analysis was performed using spectrophotometric data. The colour was measured by the bench-top spectrophotometer CM-5 Konica Minolta. The spectrophotometer was calibrated with a built-in white standard and on air. The whole analysis was performed in an xy chromaticity diagram supplemented with coordinate *Y* and CIE L*a*b* colour spaces. The ratio of the white chromophore amount to the amount of all achromatic chromophores is related to the *Y* coordinate. The ratio of the chromatic chromophore amount to all chromophores amount is saturation. The constructed model of the spruce wood colour is composed of four chromophores. The white chromophore belongs to holocellulose. The black chromophore belongs to lignin. The saturation is influenced by two chromophores. One of them belongs to extractives, another to lignin. The amounts of chromophores correlated with the spruce wood chemical composition. The chemical composition was measured using the procedures of Seifert, Wise, Sluiter, and ASTM. Moreover, the wood colour is affected by the moisture content.

## 1. Introduction

The researcher realises the wood colour in association with its change. Wood colour, together with its physical and mechanical properties, is an important quality parameter because colour is associated with decay resistance, commonly known as natural durability [1,2]. The colour of the wood is important for its users, especially for indoor wood products such as claddings, floorings, and furniture [3,4]. The colour inhomogeneity of wood generates a beautiful colour harmony [5].

The variability of wood colour can be explained in various ways at different levels of wood structure. For example, the magnitude of the surface contact area with the surroundings is inversely proportional to the intensity of reflected light. Then, free water refracts the light on the meniscus inside the cell lumen and light collides with the large wood surface with greater probability. The macrostructure of spruce wood consists of lighter earlywood and darker latewood. The wood colour differs in various wood anatomical sections as was measured by Hirata et al. [6]. The quality of the section (e.g., cross, radial, tangential) is not only a parameter of the section. The area of the captured section is significant in colour measurement.

The chemical level of the wood structure is characterized on a qualitative and quantitative basis. In addition to cellulose, hemicelluloses, and lignin, wood also contains about 2 to 5% extractives [7].

The main structural substances of wood, cellulose and hemicelluloses, do not absorb visible light [8]. However, degraded cellulose has been found to be one of the contributors to the formation of chromophores [9,10]. It results in a loss of quality of cellulosic pulp and pulp derivatives. Oxidised functional groups, such as the carbonyl moiety of aldehyde groups and conjugated diketones, are known to be responsible for the yellowing of dissolving wood pulp.

Brightness reversion due to hemicellulose content in the pulp was investigated by Beyer et al. [11]. Findings from the study showed that the yellowing mechanism is a two-step process: the first step is the decay of unstable hemicelluloses to form carbohydrate compounds, followed by dehydration and condensation of the resulting compounds, to form coloured compounds that cause brightness reversion [11].

The third main component, lignin, absorbs and reflects light below a 500 nm wavelength and has a pale-yellow colour [8]. However, Gonzáles-Peña and Hale [12] showed the brown colour of lignin. According to Falkehag et al. [13], the exact nature of the colour-causing structures of kraft lignin is uncertain, but the following chromophores may contribute to the colour: (1) CH=CH double bonds conjugated with the aromatic ring; (2) quinonemethides and quinones which also may serve as oxidative species creating further chromophoric structures; (3) chalcone structures; (4) free radicals; (5) metal complexes with catechol structures. The three latter structures are likely to contribute to the colour of kraft lignin only to a minor extent. Chemical analyses show that the deterioration of irradiated wood is primarily related to the decomposition of lignin [14,15]. Free phenoxyl radicals are created by the degradation of lignin. These free radicals react with oxygen to produce carbonyl chromophoric groups [16,17]. The chromophoric groups are partly responsible for the discolouration of the wood. Moreover, Tribulová et al. [18] cited Falkehag et al. [13] that the colour of natural light-yellow wood is most likely caused by chromophores in lignin and extractives. The chemical components determining the colour of the wood are the extractives [19]. The extractives in wood are also sensitive to light irradiation and the colour change is generated by the chromophoric degradation products [20].

The wood discolouration due to moisture content change occurs even in the range below the FSP (fibre saturation point), in most species, achieved values observable by the human eye [21]. Nevertheless, it can be neglected if the narrower range of MC (moisture content) fluctuation is considered, or the wood MC is lower than 15% [21]. In the study of Yeo et al. [22], colour change on wood surfaces treated with CoCl_2_, according to humidity was used for determining surface moisture content non-destructively and continuously during drying.

The precise modelling of the typical behaviour of material properties (i.e., colour) is not only important for exterior applications but this procedure can be used to analyse changes in material properties in interior applications (e.g., different coating systems) [23].

The results of colour measurement, as it is perceived by a human, are expressed in CIE L*a*b* colour space. The expression of the mixture of two different colours is advantageous in the chromaticity diagram because the result is placed on a straight line between these two colours [24,25].

The analysis of spruce wood colour in terms of its chemical composition is the aim of this contribution. For this purpose, the spruce wood compounds will be isolated. Then, the spruce wood colour and the colour of the wood components will be shown in the colorimetric diagram and CIE L*a*b* colour space. Later, the estimation of the moisture content effect on the *Y* coordinate and lightness will be provided. The analysis will be performed in three basic anatomical directions.

## 2. Material and Methods

**Figure 1:**
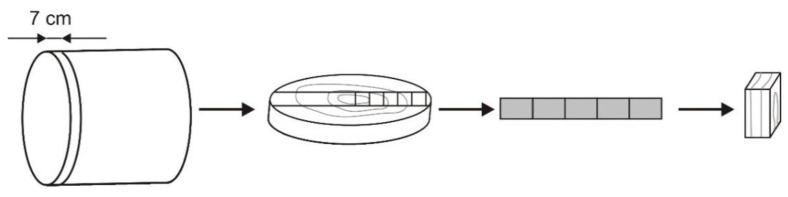
Scheme of test figure production.

The spruce wood *(Picea abies* L. Karst) was grown in Lukové (Slovakia). The spruce was felled in December at a height of 0.5 m from the earth’s surface and the sample—December—was handled at the length of 1 m from the butt cut just after felling. The total length of the roundwood was 5 m. Subsequently, three pieces of roundwood with a length of 1 m were handled apart from the sample named December. They were stored for 2, 4, and 6 months. A disc with a thickness of 7 cm was cut from the samples at the beginning of each month. Rectangle-shaped samples with dimensions of 20 mm × 20 mm × 30 mm (radial, tangential, and longitudinal anatomical directions) were cut from the disc (Figure 1). From the rest part of the disc, the sample in the shape of “V” was cut and subjected to chemical analyses.

Test figures were produced from the second, third, as well as fourth part of the roundwood. These three parts were debarked after 2, 4, and 6 months of storage before test figure production. The number of 27 was the total amount of samples.

### 2.1. Determination of Moisture Content and Colour

The moisture content was determined according to the standard STN EN 490103 [26] using the gravimetric method. Density at the given moisture content was measured according to the standard STN EN 490108 [27].

Spruce wood colour was measured using benchtop type Spectrophotometer CM-5 (Konica Minolta, Tokyo, Japan) with a wavelength resolution of 10 nm. The ambient air parameters were a temperature of 21 °C, relative humidity of 45%, and pressure of 101.3 kPa whole measured with Data logger ALMEMO^®^ 2890-9 (Ahlborn, Holzkirchen, Germany). The measurement spot diameter of 8 mm was selected on the top of the spectrophotometer according to the dimensions of the samples’ surfaces prior to colour measurement. The spectrophotometer was calibrated prior to each measurement according to the procedure recommended by the producer. The white standard is built in spectrophotometer. The whole calibration was guided by a computer using the Colour Data Software CM-S100W SpectraMagic^TN^NX (Konica Minolta, Tokyo, Japan). The reflectance spectrum was measured under illuminant D65 with specular components excluded. The 10°degree standard observer was set prior to the computation of colour coordinates. The spectrophotometer provided the three parameters of CIE L*a*b* colour space [24] and two parameters of chromaticity diagram [25] and spectrum of relative spectral intensity among others. The transmittance spectrum of dissolved extractives (concentration of extractives was 0.2 g/100 mL) was measured under illuminant D65 with specular component excluded. The colour measurement consisted of the 7 recordings for each wood anatomical section and each sample. In total, 1134 colour measurements were performed. The small clear samples were free of visual defects. The quartz glass cuvette of 10 mm thickness was used to measure the transmittance spectra. The holocellulose, cellulose, and lignin in the form of powdery samples were measured in the quartz glass beaker in a diameter of 3 cm. The samples were not pressurised, only poured from almost zero height.

### 2.2. Determination of Extractives, Holocellulose, Cellulose, Hemicelluloses, and Lignin

Samples of spruce wood were mechanically disintegrated into sawdust. Fractions of sawdust of the size 0.5–1.0 were extracted in the Soxhlet apparatus with a mixture of ethanol and toluene according to the American Society for Testing and Materials [28]. Holocellulose was determined using the procedure of Wise et al. [29], and cellulose by the Seifert method [30]. Hemicelluloses were calculated as the difference between holocellulose and cellulose. The lignin content was determined according to the NREL (National Renewable Energy Laboratory) laboratory analytical procedure [31].

All measurements were performed using three replicates per sample. The data were presented as percentages of oven-dried mass of unextracted wood.

### 2.3. Modelling of Wood Colour

The *Y* coordinate in the chromaticity diagram is regarded as a qualitative property. It is modelled using the sum rule in the form:

(1) $$Y_{L}=\frac{Y_{Holo}p_{Holo}+Y_{Lig}p_{Lig+Y_{Ext}p_{Ext}+Y_{Wat}w}}{w+p_{Holo}+p_{Lig}+p_{Ext}}$$

(2) $$Y_{R}=k_{R}\frac{Y_{Holo}p_{Holo}+Y_{Lig}p_{Lig+Y_{Ext}p_{Ext}+Y_{Wat}w}}{w+p_{Holo}+p_{Lig}+p_{Ext}}$$

(3) $$Y_{T}=k_{T}\frac{Y_{Holo}p_{Holo}+Y_{Lig}p_{Lig+Y_{Ext}p_{Ext}+Y_{Wat}w}}{w+p_{Holo}+p_{Lig}+p_{Ext}}$$

where subscripts *L*, *R*, and *T* denote cross, radial, and tangential anatomical sections; *Holo*, *Lig*, *Ext*, and *Wat* are subscripts of Holocellulose, Lignin, Extractives, and water; *k* is constant representing the ratio of contact areas with light to cross section area; *p* is a proportion of chemical substance in chemical composition the base on dry matter; *w* is moisture content based on the dry matter.

The ratio r of black chromophores amount to the number of chromatic chromophores is modelled according to a formula:

(4) $$r=\frac{\left(1-S\right)}{S}\left(1-Y\right)$$

where *Y* is the substance colour coordinate in xyY colour space, and *S* is the coordinate saturation of the substance in xyY colour space.

The matter ratio is the ratio of wood dry matter to the wood wet matter. It is a difference reminder of moisture content based on the wet matter to 100%.

## 3. Results and Discussion

**Figure 2:**
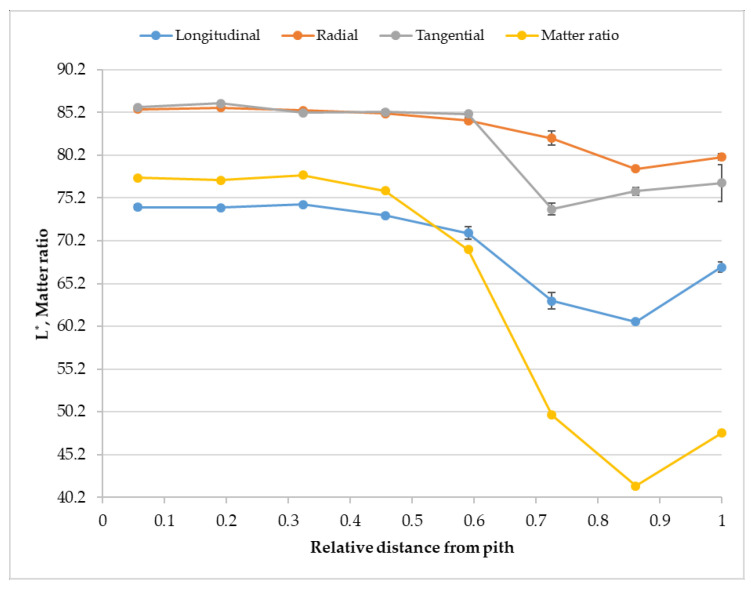
The coordinate L* in dependence on the distance from the pith.

**Figure 3:**
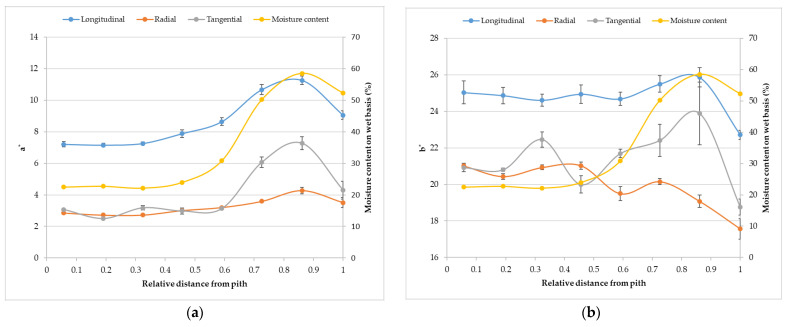
The coordinates a* (**a**) and b* (**b**) show reverses characters in dependence on the distance.

The appearance of spruce wood colour is shown in Figure 2 and Figure 3 in CIE L*a*b* colour space. The independent quantity is the distance from the pith (x-axis) and the dependent variables are lightness, a* and b* coordinates along with moisture content and matter ratio. The value of lightness is different with ascending distance from the pith.

The lightness copies the character of the matter ratio, while a* and b* copy the character of the moisture content. The literature data on spruce wood colour are shown in Table 1.

The large variability of literature data is evident in all three coordinates of CIE L*a*b* colour space. Mostly, the variability is attributed to the measured density of the wood. The measured oven-dried density was 443 kg·m^−3^ in mean values with a standard deviation of 38 kg·m^−3^.

Using the xyY colour space is more convenient for the purpose of modelling a spruce wood colour. The mix of colours is between the mixing colours and is located in a straight line between them. Table 2 contains mean values and standard deviations of moisture content based on dry matter and colour coordinates of xyY and CIE L*a*b* colour spaces at a given moisture content and at different times of sample preparation. Table 2 shows a large variability of data in the form of standard deviation. The source of the variability is found mainly in the moisture content and anatomical section. The different times of producing specimens reflect the change in moisture content.

The chemical composition is another parameter of spruce wood colour variability.

Table 3 shows a small variability of chemical composition with the increasing height of the stem in different times of specimen preparation.

Table 4 shows the statistical characteristics of colour coordinates of xyY and CIE L*a*b* colour spaces of spruce wood compounds.

**Figure 4:**
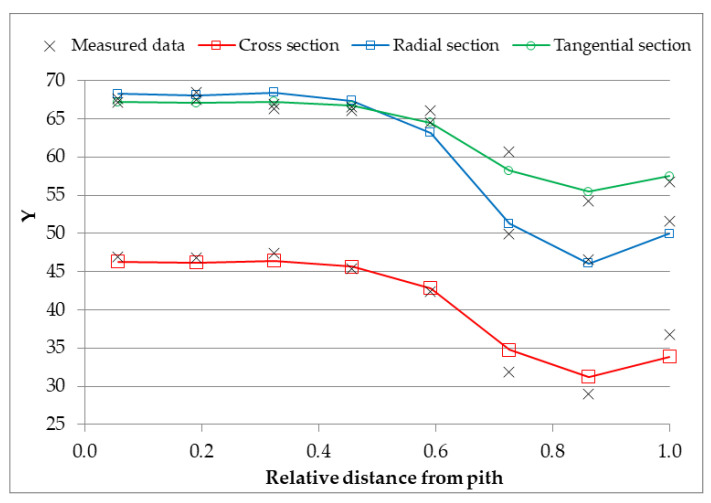
The measured data of the *Y* spruce wood colour coordinate and its theoretical representative on different anatomical sections.

Chemical compositions together with compound colour are mixed to match wood colour using Formulas (1)–(3). Figure 4 shows the ability of formulas to predict the *Y* coordinate.

Table 5 shows the computed *Y* coordinates of spruce wood in different anatomical sections using the least square method. The standard deviation of computed to measured values of *Y* was 1.8 in the Cross or Radial sections and 1.1 in the Tangential section.

The *Y* coordinate characterizes the achromatic chromophores, and its value is the ratio of the white chromophore amount to all the achromatic chromophores amount. The *Y* of holocellulose does not well match its measurement, in cross and radial sections. It is assumed that the extractive chromophore is located on a curve of spectral colours, and it does not contribute to achromatic chromophores. Water is characterized by different values of the *Y* coordinate in cross or radial and tangential sections. The difference between measured and computed *Y* values of water can be assigned to existing aqueous solutions of substances in freshly cut wood. Another source of mistakes is in different surface moisture contents of samples which are not in equilibrium with the surroundings. However, equilibrating the samples with surroundings is a long-time process during which many other factors, such as radiation, heat, force, and others, influence the results.

The calibration curve between the moisture content of spruce wood and its *Y* coordinate has the form:

(5) $$Y_{L}=\frac{58.67+13.1\frac{w}{100}}{1+\frac{w}{100}}$$

(6) $$Y_{R}=1.475\frac{58.67+13.1\frac{w}{100}}{1+\frac{w}{100}}$$

(7) $$Y_{T}=1.801\frac{43.66+23.0\frac{w}{100}}{1+\frac{w}{100}}$$

where *w* is moisture content based on dry matter in percentage. The relationships (5)–(7) reveal the anisotropic character of wood which is hardly influenced only by chemical composition. The indexes of correlations are 87.2%, 81.1%, and 84.1%.

**Figure 5:**
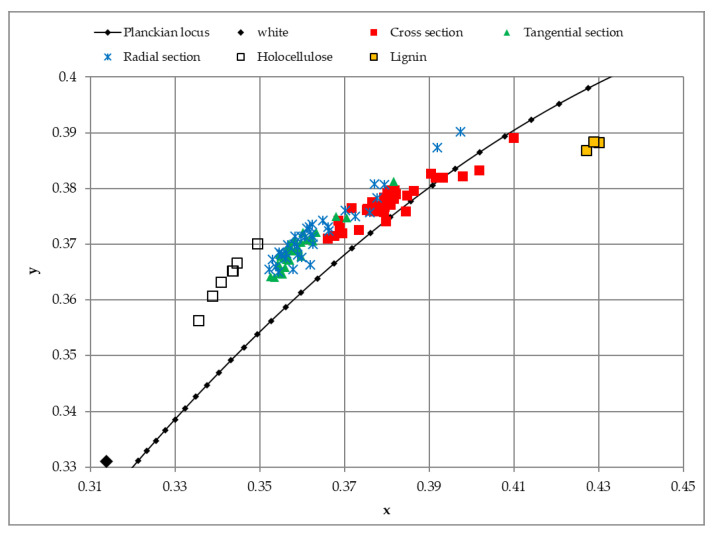
Spruce wood and its chemical compounds in chromaticity diagram.

Figure 5 shows spruce wood and its chemical compounds in a chromaticity diagram.

Spruce wood colour on the cross, radial, and tangential sections is located along the Planckian locus (Color Data Software CM-S100W, Konica Minolta, Tokyo, Japan) and between holocellulose and lignin colours, Figure 5.

Following the data in Table 4, it can be stated that the coordinate saturation of lignin is *S* = 0.48. Then, the ratio of the black lignin chromophores amount to chromatic lignin chromophores amount is 1.0. It means that lignin contains equal amounts of chromatic and black chromophores. As far as extractives chromophores are only chromatic and extractives do not contain black chromophores, the saturation of wood colour reveals that the chromaticity of wood is influenced by extractives and with lignin chromatic chromophores. It is because the percentage of extractives is too small to compose the wood chromaticity alone. To secure wood colour saturation from outside influences, it is necessary to protect both extractives and lignin chromophores. Otherwise, one can expect the pale wood colour which is mainly composed of holocellulose and lignin achromatic chromophores.

## 4. Conclusions

The analysis of spruce wood colour measurement revealed the impossibility of determining wood colour only by the summation of information from the measurement of isolated wood compounds. The colour of wood in different anatomical sections depends on the other information. We assumed that the area where light collides with wood plays an important role in their interaction. The appearance of the spruce wood colour was shown in the CIE L*a*b* colour space. Spruce wood is darker, more saturated, and shifted to red if the moisture content is ascending. Holocellulose is the lightest and is mostly compounds with white chromophores. The presence of a small number of extractives coloured its toluene-ethanol solution intensively. When the chromophore of extractives is treated as fully saturated, then the saturation of wood cannot be explained only by them, because of a small number of extractives in the wood. Therefore, lignin must contain achromatic black as well as chromatic chromophores. Their ratio is almost 1. The inhomogeneous spruce wood colour depicted in the chromatic chart followed the Planckian locus direction.

## Figures and Tables

**Table 1:** The mean values of density and colour parameters L*, a*, b*.

	Density (kg·m^−3^)	L* (Std. Dev.)	a* (Std. Dev.)	b* (Std. Dev.)
Oberhofnerová et al. [32]	533 (-)	84.16 (0.16)	3.72 (0.08)	19.54 (0.20)
Torniainen et al. [33]	-	82 (1.2)	4 (0.9)	19 (1.7)
Geffertová et al. [34]	-	82 (-)	2.6 (0.5)	17.3 (0.7)
Humar et al. [35]	432 (10)	83.8 (1.3)	6.5 (0.4)	10.4 (0.4)

**Table 2:** Statistical characteristics of moisture content based on the dry matter and colour coordinates of spruce wood.

			*w*	*Y*	*x*	*y*	L*	a*	b*
Cross section	December	Average	0.642	40.94	0.3863	0.3796	69.83	8.71	24.86
		Std. Dev.	0.425	6.89	0.0080	0.0022	5.14	1.52	1.03
	February	Average	0.622	42.68	0.3808	0.3771	70.85	7.68	23.32
		Std. Dev.	0.580	9.69	0.0156	0.0064	6.66	2.09	2.43
	April	Average	0.647	44.03	0.3753	0.3764	72.03	6.33	22.64
		Std. Dev.	0.517	6.95	0.0091	0.0037	4.45	1.44	1.42
	June	Average	0.390	41.20	0.3795	0.3775	70.25	7.31	23.28
		Std. Dev.	0.226	3.73	0.0060	0.0034	2.48	0.71	1.47
Tangential section	December	Average	0.642	63.43	0.3577	0.3688	83.60	3.25	19.91
		Std. Dev.	0.425	4.82	0.0015	0.0018	2.64	0.53	1.20
	February	Average	0.622	60.21	0.3621	0.3712	81.53	3.86	20.89
		Std. Dev.	0.580	12.04	0.0102	0.0050	6.55	1.53	1.80
	April	Average	0.647	60.68	0.3591	0.3691	82.12	3.64	19.95
		Std. Dev.	0.517	5.74	0.0042	0.0031	2.97	0.53	1.83
	June	Average	0.390	64.19	0.3572	0.3672	84.06	3.66	19.24
		Std. Dev.	0.226	4.24	0.0055	0.0040	2.06	0.60	1.89
Radial section	December	Average	0.642	61.26	0.3626	0.3713	82.24	4.07	21.27
		Std. Dev.	0.425	8.87	0.0070	0.0028	5.07	1.66	1.76
	February	Average	0.622	57.47	0.3703	0.3761	79.87	4.91	23.36
		Std. Dev.	0.580	13.75	0.0186	0.0100	7.57	2.58	4.37
	April	Average	0.647	56.02	0.3662	0.3730	79.16	4.65	21.78
		Std. Dev.	0.517	12.51	0.0100	0.0057	6.77	1.63	2.89
	June	Average	0.390	61.74	0.3590	0.3681	82.72	4.02	19.74
		Std. Dev.	0.226	4.85	0.0048	0.0030	2.41	0.88	1.85

**Table 3:** Chemical composition of spruce wood.

*p* (%)		December	February	April	June
Holocellulose	Average	79.22	79.13	78.16	77.17
	Std. Dev.	1.25	0.44	2.04	0.58
Cellulose	Average	44.05	44.18	44.07	43.69
	Std. Dev.	0.98	0.51	0.40	1.97
Hemicelluloses	Average	35.17	34.95	34.09	33.48
	Std. Dev.	0.6	0.95	2.43	2.55
Lignin	Average	25.42	24.83	24.73	24.25
	Std. Dev.	0.57	0.10	0.30	0.02
Extractives	Average	1.34	1.25	1.22	1.38
	Std. Dev.	0.08	0.03	0.05	0.05

**Table 4:** The measured colour coordinates in xyY and CIE L*a*b* colour spaces of spruce wood compounds.

		*x*	*y*	*Y*	L*	a*	b*
Holocellulose	Average	0.342	0.363	51.41	76.889	−1.015	14.211
	Std. Dev.	0.005	0.005	3.23	1.936	0.303	2.324
Cellulose	Average	0.352	0.364	43.84	72.062	2.576	15.304
	Std. Dev.	0.014	0.009	3.76	2.504	1.906	4.439
Lignin	Average	0.429	0.388	6.28	30.103	10.423	19.003
	Std. Dev.	0.002	0.001	0.23	0.584	0.225	0.524
Extractives *	Average	0.327	0.349	88.96	95.56	−1.58	9.05
	Std. Dev.	0.006	0.007	1.67	0.70	0.44	3.72
Water	Average	0.3137	0.3312	92.35	96.96	−0.14	0.04

* Saturated solution in toluene ethanol.

**Table 5:** The computed *Y* coordinates of spruce wood on different anatomical sections.

	Cross and Radial Sections	Tangential Section
	*Y*	*Y*
Holocellulose	70.6	51.4
Lignin	6.30	6.21
Water	13.1	23.0

## Data Availability

The data presented in this study are available on request from the corresponding author.
